# Empathy and Burnout – A Cross-Sectional Study Among Mental Healthcare Providers in France

**DOI:** 10.25122/jml-2018-0050

**Published:** 2019

**Authors:** Livia Sturzu, Adrian Lala, Michael Bisch, Marie Guitter, Daniela Dobre, Raymund Schwan

**Affiliations:** 1. Pôle Hospitalo-Universitaire de Psychiatrie d’Adultes du Grand Nancy, “Centre Psychothérapique de Nancy”, Laxou, France; 2. Centre Hospitalier Spécialisé de Sarreguemines, Sarreguemines, France; 3. Centre Hospitalier de Sarreguemines, Hôpital “Robert Pax”, Service des Urgences, Sarreguemines, France; 4. “Centre Psychothérapique de Nancy”, Laxou, France; 5. INSERM U1114, Fédération de Médecine Translationnelle de Strasbourg, Pôle de Psychiatrie, Centre Hospitalier Régional Universitaire de Strasbourg, Strasbourg, France; 6. Maison des Addictions, CHRU Nancy, France; 7. University Lorraine, Faculty of Medicine, Department of Psychiatry, Vandoeuvre-Les-Nancy, France

## Abstract

Previous studies have established a correlation between empathy and burnout among healthcare providers. The aim of this study is to explore whether empathy – the ability to understand what another person is experiencing, was related to mental healthcare staffs’ burnout.

We performed a descriptive, cross-sectional, observational study among medical and nursing mental healthcare staff working in the district of Moselle, France. Participants completed a survey including The French versions of the Jefferson Scale of Empathy (JSPE) and The Maslach Burnout Inventory-Human Services Survey (MBI-HSS).

The sample included 241 (n=241) participants (N=420, response rate=51.7%). 187 (77.6%) respondents had low burnout, 43 (17.8%) had intermediate burnout and 11 (4.6%) had high burnout. 41 (17%) had low empathy, 156 (64.7) had moderate empathy and 44 (18.3%) scored high.

Empathy scores were positively correlated with scores of personal accomplishment of the MBI-HSS (r=0.2; p<0.001), but negatively correlated with scores of depersonalization (r=-0.2; p<0.003). Highest means of depersonalization (DP) (M=8.7; SD=6.8; p<0.009) and lowest means of compassionate care (M=40.05; SD=7.9; p=0.0001) were found among forensic psychiatric security units staff.

Participation in the Omega educational program was associated with lower scores of EE on the MBI-HSS survey (mean score 14.7 versus a mean score of 19.7 for nonparticipants).

Empathic mental healthcare providers have lower levels of burnout. Forensic psychiatric staff showed low means of compassionate care and high depersonalization.

Interventions designed to foster attributes and skills such as empathy, resilience, and perception of security may be an essential step in reducing and preventing burnout.

## Introduction

The dynamics of recent healthcare reforms and the pervasive economic aspects changed conceptions of mental health professionals. In addition to organizational stressors common to all human services workers, they face additional emotional strains due to the stigma and misunderstanding of mental illness.

In France, “the psychiatry crisis” has been a pressing topic in 2018 with a reported increase of 60% in need for treatment in the last ten years as well as a reduction in the number of caregivers, that geographically varies between 8% and 47% [1]. The shortage of mental healthcare resources is problematic, with an average rate of vacant jobs of 28.7% for psychiatrists, which means a reduction in the time doctors can allocate to every patient [2].

Difficulties in adapting to novel demand cause psychological distress which may be responsible for burnout and consequently absenteeism, low organizational commitment and poor job performance [3–5].

Further research has shown that burnout is likely to have a contagion effect, progressively damaging the morale of employees and leading to further staff turnover [6].

Also, the doctor-patient relationship has undergone dramatic changes in recent years due to increasing access to information, patient-centered care and social changes that increased patient autonomy [7]. In response to these changes, the medical community has realized the importance of both augmenting awareness of the impact of doctors’ attitudes on clinical outcomes, patient satisfaction, and encouraging a culture of mentoring communication skills, resilience, and empathic engagement [8,9].

Empathy cultivates a deeper interpersonal relationship. It is often confused with related concepts such as compassion and sympathy. However, long-term exposure to emotionally-demanding situations may put healthcare providers at risk for burnout that is related, but a different construct.

The term of professional exhaustion, initially described in 1959, was later renamed as “burnout” by J.H Freudenberger [10]. The burnout syndrome has a multifactorial origin. It is defined as high levels of emotional exhaustion and depersonalization (negative or cynical attitudes), along with a diminished sense of personal achievement [11].

Burnout has been associated with negative consequences for mental health providers, including physical health problems (e.g., insomnia, headaches, poor overall health), relationship issues, reduced job satisfaction, and increased mental health problems (e.g., depression, anxiety, substance abuse) [12].

Numerous studies have investigated the causes of burnout and proposed mechanisms to reduce this state of emotional, mental, and physical exhaustion [13]. Staff burnout is widely believed to impact the quality of care because of the decreased effectiveness which ultimately leads to poorer treatment outcomes [14].

Measuring the quality of care is especially difficult in mental health services because of a scarcity of measurement tools and that of consistent infrastructure and strategy for assessing quality [15]. In this context, workers’ feelings of job disaffection and burnout can play a crucial role in further reducing the mental health service workforce.

Empathy has been described as the royal road to an optimal physician-patient relationship, and an essential component of overall physician competence [16]. The concept is illustrated as the ability to understand the experiences and feelings of another person and to communicate this understanding to them [17]. It is widely recognized as a protective factor against burnout. Previous data suggested a positive association between caregivers’ empathy and patient satisfaction [18]. An empathic caregiver is believed to reduce patients’ anxiety and improve clinical outcomes [19]; this, in turn, might enhance the sense of usefulness among healthcare providers and ensure better job satisfaction.

In social-cognitive neurosciences, empathy is frequently defined as the capacity to feel and share the emotions of others. Therefore, empathy is described as an affective state and emotional reactions that are caused by emotional sharing [20].

The current growing interest for empathy in medicine contrasts with a form of “detached concern” that has been preached in the 1960s and has long been considered as essential to the care relationship. When healthcare providers aim for an idealized version of empathy by suppressing personal emotions and yet motivating the quality of care with a “detached concern”, they aim to protect themselves against burnout or, more specifically, compassion fatigue. Objectivity is seen as crucial for making tough diagnoses, and stoicism is believed to be necessary for providing invasive, sometimes noxious, treatment [21].

In 1906, W. Osler had already defined the neutralization of emotions as the necessary condition for physicians “to see into” their patients and access “their interior life”. According to this approach, the relationship towards patients is intellectualized and excludes any affective dimension [21].

Although research on work-related stress in the healthcare sector is substantial, little attention has been directed towards reducing or preventing burnout among mental health professionals. This is both surprising and ironic, given the goals of mental health organizations for improving the behavioral health of individuals. The need for burnout prevention and interventions for mental health providers has been highlighted by researchers for decades, but few such programs have been implemented and evaluated.

The primary objective of our study was to describe how patterns of empathy are associated with mental health care staffs’ burnout. We hypothesized that a higher level of burnout would affect empathy as previously shown in recent studies conducted among other healthcare professionals [22–24]. As secondary objectives, we aimed to identify socio-demographic, job-related factors and education and training programs that may impact clinical empathy among mental healthcare providers.

## Materials and Methods

### Study design

We conducted a descriptive, cross-sectional, observational study among mental healthcare providers (psychiatrists, psychiatry trainees, mental health nurses) working in the district of Moselle, a province in north-eastern France. The district has three psychiatric hospitals that serve a population of approximately 560.000 inhabitants. Psychiatric hospitals are organized on a regional basis and integrated with the general hospital system. Each ward team is responsible for a geographic section of the district. One hospital included a forensic psychiatric unit. Participants were selected on a voluntary basis. Healthcare professionals were contacted using the professional email and asked to voluntarily fill out an anonymous survey between October and December 2017.

### Instruments

The survey included socio-demographic and professional context data, the French version of the Jefferson Scale of Physician Empathy (JSPE) and the Maslach Burnout Inventory-Human Services Survey (MBI-HSS).

Subjects provided basic socio-demographic and professional context data, collected using a survey-like questionnaire. The collected data included: sex, age, marital status, number of children, urban/rural divide, professional status (psychiatrist, psychiatry trainee, chief nurse, nurse) average number of weekly work hours, personal experience with the Omega training program, which is an education and training program used to teach healthcare and mental health workers the skills needed to effectively intervene in situations of aggression [25]. This program was implemented in France in 2005 and is recommended for high-risk workers to prevent workplace violence. The Omega program is taught by peer trainers (security agents). It lasts four days and seeks to teach participants the skills and intervention methods necessary to ensure their safety and that of their patients in situations of aggression [26].

The French version of the Jefferson Scale of Physician Empathy (JSPE) was used [22]. JSPE is a 20-item self-reported questionnaire assessing levels of empathy in the patient-care context. The original JSPE presents a three-factor structure: Perspective Taking (PT), Compassionate Care (CC), and Standing in Patient’s Shoes (SIPS). Participants are asked to report their level of agreement with each item in a seven-point Likert-type scale, where 1 = “Strongly disagree” and 7 = “Strongly agree”. Ten items are positively worded, and ten items are negatively worded. Three partial scores (PT, CC, and STS) and one global score may be calculated (by the sum of its corresponding items), with higher scores (ranging from 20 to 140 for the total scale) reflecting a higher attitude towards empathy [17]. Internal consistency was estimated by Cronbach’s coefficient alpha. The reliability coefficients for the subscales were calculated: 0,752 for Perspective Taking for 0,674 for Compassionate Care and 0,679 for Standing in Patient’s Shoes.

In the initial validation study for the French version of the JSPE, compassionate care was moderately correlated to scores for standing in patient’s shoes and perspective taking (r = 0.46 and r = 0.33, p < 0.01, respectively), while perspective taking was moderately correlated to standing in patient’s shoes (r = 0.17, p < 0.01). These results confirmed the absence of a general physician empathy factor for the present sample. Consequently, the following analyses only considered scores for the three independent subscales [23].

Burnout was measured using the French version of the 22-item Maslach Burnout Inventory-Human Services Survey (MBI-HSS), which has been formally validated and used in previous studies [27]. The instrument comprises 22 items, which are divided into three subscales: emotional exhaustion (EE, 9 items intended to measure emotional exhaustion, possible score range 0-54), depersonalization (DP, 5 items intended to measure perceptions of impersonal, non-appreciative responses by others for providing services or help, possible score range 0-30), personal accomplishment (PA, 8 items, measuring perceptions of competence and successful academic and professional achievement, possible score range 0-48). The items are written in the form of statements about personal feelings or attitudes (e.g., ‘I feel burned out from my work,’ ‘I do not really care what happens to some recipients’). The items are answered in terms of the frequency with which the respondent experiences these feelings, on a 7–point, fully anchored scale (ranging from 0, ‘never’ to 6, ‘every day’) [28].

A high level of emotional exhaustion is defined as a score >27, and a high level of depersonalization is defined as a score >13. Low personal accomplishment is defined as a score <31 [27]. High burn-out was defined as a high score on the emotional exhaustion subscale, a high score on the depersonalization subscale and a low score in personal accomplishment. Intermediate burnout was defined as high scores of emotional exhaustion and, either high scores of depersonalization or low scores of personal accomplishment. For the purpose of the current study, scores within individual burnout domains were used both as continuous variables and categorized into low, intermediate and high scores using the cutoffs suggested by Maslach et al.[28].

The reliability coefficients for the MBI–HSS were based on samples that were not used in the item selections to avoid any improper inflation of the reliability estimates. Internal consistency was estimated using Cronbach’s coefficient alpha. The reliability coefficients for the subscales were calculated: 0.905 for Emotional Exhaustion, 0.705 for Depersonalization, and 0.707 for Personal Accomplishment. The results were similar to the original validation study [28].

### Data collection

Data were collected using two procedures: 203 participants completed a materialized version of the questionnaire whereas 99 completed a computerized version. Overall, 420 subjects were prompted. 61 incomplete forms were excluded from the study, accounting for 241 retained forms in the final database (n=241). Participants answered questionnaires in the following fixed order: socio-demographic and practice survey; JSPE-Fr and MBI-HSS. The collected data were anonymous.

Informed consent and data analysis privacy issues were treated in conformity with the guidelines of the national Data Protection Commission (CNIL) of France.

### Inclusion criteria

Subject selection was made based on the work-field uninterrupted experience of at least one year in a psychiatric unit. Study participants came from three psychiatric hospitals in the Moselle Department of Eastern France with similar workforce and daily patient loads statistics.

### Statistical analysis

For data analysis, descriptive means of frequency, percentages, and standard deviations were calculated using IBM SPSS © version 16.0.

## Results

### Demographics

A total of 420 subjects were prompted from which 302 agreed to participate. Two hundred forty-one (response rate = 51.9%) were included in the present study (115 women and 126 men). The sample was composed of 161 nurses, 30 chief nurses, 30 psychiatry residents, and 20 registered psychiatrists. The average age was 39.77 (SD=11.258; 22-62 years).

Table 1 summarizes the characteristics of the sample and their scores on the MBI. We have found that 42 subjects (17.4%) scored high on EE, 44 (18.3%) scored high on the DP subscale, and 105 (43.6%) had low scores of PA.

**Table 1:** Baseline characteristics of participants and means of MBI-HSS subscales value

Variables	N (%) N =241	Burnout (mean. SD)		
		EE	DP	PA
**Gender**				
Male	126 (52)	16.4 (12)	7.9 (6)*	33.9 (6.13)
Female	115 (48)	18.3 (11)	6.3 (5)	33.9 (6.7)
**Marital status**				
Single	42 (17)	16.3 (10)	7.9 (6)	34.04 (6.2)
Couple	87 (36)	16.7 (12)	7.6 (5)	33.5 (6.6)
Married	90 (37)	18.6 (12)	6.6 (6)	34.6 (6.2)
Divorced	22 (9)	16.9 (12)	6.3 (6)	32.5 (6.4)
**Residence**				
Urban	87 (36)	18.3 (11.9)	7.3 (5.7)	34.5 (5.9)
Rural	154 (64)	16.7 (11.1)	7.05 (5.7)	33.6 (6.6)
**Profession**				
Psychiatric Nurse	161 (67)	16.5 (10.9)	7.01 (5.66)	34.1 (6.6)
Chief nurse	30 (12)	17.06 (11.4)	5.9 (5.1)	33.5 (4.5)
Resident	30 (12)	20.6 (13.7)	8.8 (6.2)	33.1 (7.1)
Psychiatrist	20 (8)	19.8 (10.5)	7.6 (5.6)	34.7 (5.7)
**Sector of practice**				
Inpatient	134 (56)	17.3 (10.2)	6.7 (4.9)*	34.05 (6.7)
CMP	37 (15)	17.6 (11.5)	5.5 (5.06)	35.02 (5.3)
UMD/USIP	70 (29)	17.1 (13.5)	8.7 (6.8)	33.2 (6.1)
**Omega program**				
Yes	114 (47)	14.7 (11)*	6.3 (5.6)*	34.4 (6.1)
No	127 (53)	19.7 (11.3)	7.8 (5.6)	33.5 (6.6)

PT-Perspective Taking, CC-Compassionate Care, SIPS-Sitting in Patient’s Shoes, JSPE- Jefferson Scale of Physician Empathy, MBI-HSS-Maslach Burnout Inventory-Human Services Survey, EE-Emotional Exhaustion, DP- Depersonalization, PA-Personal Accomplishment, CMP-Outpatient psychiatric clinic, UMD-Unité de Malades Difficiles (Forensic psychiatric security unit), USIP-Unité de soins intensifs psychiatriques (Psychiatric intensive care unit)

The mean weekly workload was 37.6 hours (SD=5.8; CI=95%). Highest workloads were registered for psychiatric trainees with a mean at 45.17 (SD=8.23; CI=95%). The variability of work hours for this group may be explained by the night shifts and weekends.

There was no significant difference in workloads stratified by inpatient or outpatient practice (mean=37.6; SD=5.8; CI=95%).

The most experienced professionals were the chief nurses with a mean of 25 years of practice (SD=11.62, CI=95%) and as expected, psychiatric trainees had the lowest professional experience.

### Variables associated with burnout and clinical empathy

Mean scores and standard deviation of the JSPE were in the range found in most other studies performed among other healthcare professionals [22,23,29]. The mean scores for emotional exhaustion, depersonalization, and personal accomplishment of the MBI-HSS scale were all in the range of moderate results for those scales [28].

The JSPE and MBI scores were grouped into three categories - low, intermediate, and high. According to the overall MBI scores in our sample, 187 (77.5%) respondents had low burnout, 43 (17.8%) had moderate burnout, and 11 (4.6%) had high burnout. Forty-one responders (17%) had low empathy, 156 (64.7) had moderate empathy, and 44 (18.3%) scored high on the empathy scale. Table 2 summarizes empathy and burnout scores.

**Table 2:** Means, Standard Deviations of JSPE and MBI-HJSS dimensions calculated for the whole study sample.

Variables	Mean (SD)
Empathy (JSPE total score)	110.3 (12.5)
PT	57.1 (7.51)
CC	43.8 (6.67)
SIPS	9.39 (2.99)
Burnout (MBI-HSS)	58.5 (14.5)
EE	17.3 (11.4)
DP	7.16 (5.6)
PA	34.05 (6.4)

PT-Perspective Taking, CC-Compassionate Care, SIPS-Sitting in Patient’s Shoes, JSPE- Jefferson Scale of Physician Empathy, MBI-HSS-Maslach Burnout Inventory Human Services Survey, EE-Emotional Exhaustion, DP-Depersonalization, PA-Personal Accomplishment

The MBI-HSS dimensions differ according to socio-demographic aspects (as described in Table 3). We found a positive association between depersonalization scores and weekly workload (r=0.06; p=0.3) and a negative association with age (r=-0.2; p=0.0001) and years of clinical experience (r=-0.2; p=0.001). The results remained significant in linear regression models for weekly workload and age.

**Table 3:** Bivariate correlations between socio-demographic variables and MBI-HSS and JSPE subscales using Spearman’s “rho” correlation coefficient with 95% confidence interval.

	Mean (SD; range)	EE rho(p)	DP rho(p)	PA rho(p)	PT rho(p)	CC rho(p)	SIPS rho(p)	Total JSPE rho(p)
Age (years)	39.7 (11.2; 22–62)	0.06 (0.3)	–0.2 (0.001)	0.1 (0.1)	0.004 (0.1)	–0.04 (0.4)	0.011 (0.8)	–0.008 (0.9)
Children (no)	1 (0.87; 0–2)	0.04 (0.4)	–0.09 (0.1)	–0.01 (0.8)	–0.1 (0.1)	–0.09 (0.2)	–0.04 (0.5)	–0.1 (0.07)
Experience (years)	13.4 (11.03; 0–42)	0.1 (0.01)	–0.1 (0.03)	0.025 (0.6)	–0.05 (0.4)	–0.08 (0.2)	–0.004 (0.1)	–0.6 (0.3)
Weekly workload (hours)	37.6 (5.8; 16–64)	0.10 (0.1)	–0.06 (0.3)	0.05 (0.4)	0.04 (0.4)	0.2 (0.01)	0.2 (0.001)	0.1 (0.06)

PT-Perspective Taking, CC-Compassionate Care, SIPS-Sitting in Patient’s Shoes, JSPE- Jefferson Scale of Physician Empathy, MBI-HSS-Maslach Burnout Inventory Human Services Survey, EE-Emotional Exhaustion, DP-Depersonalization, PA-Personal Accomplishment

Depersonalization (DP) was found to be significantly associated with the sector of practice with the highest means of depersonalization among forensic psychiatric security units’ staff (M=8.7; SD=6.8; p < 0.009).

Also, in bivariate analysis, the scores on the emotional exhaustion scale were negatively correlated by Spearman’s correlation coefficient (r) with those of personal accomplishment of the MBI-HSS (r=-0.2; p=0.002). Scores of emotional exhaustion and depersonalization were positively correlated (r=0.5; p=0.0001). Finally, the lowest scores of emotional exhaustion (EE) were significantly associated with Omega training program participation (M=14.7; SD=11; p=0.001 compared to an M=19.7; SD=11.3; p=0.001 for nonparticipants).

When examining the associations between the Jefferson Scale of Physician Empathy dimensions and socio-demographic aspects, it resulted that being a woman is associated with superior levels of compassionate care (M=45.7; SD=5.4 for women vs. M=42.01; SD=7.1 for men; p=0.001). The other dimensions did not differ according to gender.

Also, the urban divide seemed to be associated with higher scores of empathy for the three dimensions of the scale. We observed a significant difference for empathy (p=0.0001) based on urban division: higher empathy scores were associated with an urban setting (M=114.6; SD=11.7) compared to a rural division (M=107.8; SD=12.3).

Lower scores of empathy were identified among psychiatric nurses (M=107.9; SD=12.5; p= 0.001). We also noted diminished compassionate care in forensic psychiatric security units (M=40.05; SD= 7.9; p=0.0001).

### Associations between burnout and empathy among mental healthcare staff

In bivariate analysis (Table 4), scores indicative of lower burnout were associated with high PT (r=-0.2; p=0.01) and high SIPS values (r=-0.2; p=0.02). Empathy scores were significantly and positively correlated with scores of personal accomplishment of the MBI-HSS (r=0.2; p < 0.001), but inversely correlated with scores of depersonalization (r=–0.2; p <0.003). Also, scores on the personal accomplishment scale were correlated positively with those of perspective taking (r=0.3; p <0.001) and SIPS (r=0.2; p=0.013) of the JSPE scale. This association remained significant in the linear regression model.

**Table 4:** Bivariate correlations by Spearman’s rho test for MBI-HSS and JSPE subscales on a 95% confidence interval for the whole study sample.

Spearman’s rho (p value), 95% CI	PT	CC	SIPS	JSPE total score
EE	0.009 (p=0.8)	0.09 (p=0.1)	–0.5 (p=0.4)	0.04 (p=0.5)
DP	–0.1 (p=0.074)	–0.2 (p=0.006)	–0.2 (p=0.004)	–0.2 (p=0.003)
PA	0.3 (p=0.001)	0.09 (p=0.2)	0.2 (p=0.013)	0.2 (p=0.001)
MBI HSS Total	–0.2 (p=0.01)	0.001 (p=0.9)	–0.2 (p=0.02)	–0.1 (p=0.06)

PT-Perspective Taking, CC-Compassionate Care, SIPS-Sitting in Patient’s Shoes, JSPE- Jefferson Scale of Physician Empathy, MBI-HSS-Maslach Burnout Inventory-Human Services Survey, EE-Emotional Exhaustion, DP-Depersonalization, PA-Personal Accomplishment

## Discussion

To our knowledge, this is the first study to assess the association between low empathy and burnout in mental healthcare providers in France.

It is generally believed that mental health workers have a higher risk of burnout compared to their colleagues in other medical specialties [30]. In our study, while a fifth of the participants had high empathy according to the JSPE, just 4.6 % had high burnout scores on the MBI. However, burnout rates were increased on the personal accomplishment subscale (43.6%). Methodological differences might explain the relatively small percentage of reported burnout in our sample. Different cutoffs are used for the MBI-HSS scale, and many interpretations are available.

The challenging demands on mental healthcare staff can lead to stress and frustration, that in turn, can fuel the exhaustion, cynicism, and inefficacy. Violent incidents with patients can be emotionally draining and difficult to manage and can lead health providers to distance themselves from their work psychologically. The occurrence of violence can also make providers feel that they lack control over their job, and thus challenge their sense of professional efficacy [31].

A positive correlation was observed between high depersonalization and workload and a negative correlation with age. Our results are consistent with the current research in burnout [32]. While most efforts to prevent burnout are focused on improving individual attributes, like resilience, we feel that more resources should be directed towards reducing administrative tasks that increase workload, in favor of caregiver-patient relationship.

The results of the study supported our initial hypothesis that there is a robust association between empathy and burnout among mental healthcare providers. In bivariate statistical analysis, empathy is positively correlated with personal accomplishment but negatively correlated with the depersonalization scale as shown in previous research [28].

The fact that scores indicative of lower burnout were associated with higher perspective taking and that scores of emotional exhaustion were negatively correlated with perspective taking is in line with previous studies that used the JSPE [23,33,34]. We found no significant differences between men and women regarding the levels of burnout. In our sample, women were found to have more compassionate care, as suggested in previous studies [23, 35–37].

Empathy is typically seen as an essential component of nursing care [38]. Contrary to previous findings, in our study, nursing staff had the lowest scores of empathy on the three dimensions of the JSPE. This decline in empathic behavior may be explained by a perceived lack of organizational support due to the decreased availability of resources in recent years. We may also imply that a lack of support from supervisors and medical staff could play a key role in explaining our results.

Finally, as evidence suggests, empathy varies according to the type of emotion that is expressed by patients [39,40]. In mental healthcare, patients might be very challenging as they are often prone to show aggressive behavior which may decrease empathic responses from nursing staff.

Among the unexpected results, we found that subjects working in forensic psychiatry security units reported high depersonalization and low compassionate care. The emotional distance between staff and patients might be considerate as a “necessary deficiency” in high-security wards to reduce the emotional impact of violence towards healthcare providers. The low compassionate care scores might be explained by the internal functioning of these centers, that require transfer to a center with reduced security level as soon as the patients are considered receptive to treatment. This may diminish staffs’ perception of completeness of their mission. We can also speculate that compassionate care, considered as “affective empathy”, is influenced by the process of arousal and thus could be harmful when dealing with aggressive patients.

Psychosocial intervention programs might be needed to enhance attributes like compassionate care and thus prevent burnout. Mindfulness-based stress reduction (MBSR) seems to increase (job-related) personal accomplishment and decrease emotional exhaustion and total MBI scores [41]. Stress management training should be provided in nursing and medical school, with job-specific skills implemented later in specialty training.

Developing skills to deal with aggression effectively could improve coping mechanisms and resilience capacities for mental healthcare providers. For instance, we found that previous experience with the Omega education and training program was strongly associated with lower levels of emotional exhaustion and total burnout score on the MBI scale. These results are consistent with previous research, which established a positive association between workplace violence and burnout [42–44]. In addition, being confronted to patients’ hostile behavior is associated to a lesser level of confidence in a person’s own abilities to cope with patient aggression [45], or it is well known that mental healthcare staff, given the high risk of violence, needs to be confident when working with aggressive patients [44].

The Omega education and training program was found to have a positive and significant impact on burnout, the perception of security, and the level of exposure to violent acts among mental care providers [25,26,46]. Therefore, we suggest that this program should become a mandatory part of the training in a psychiatric setting rather than optional as it is now.

In our study, empathy levers are higher among urban divide caregivers. We can speculate that the lower empathy scores observed in the rural setting reflect the escalating caregiver shortage in those areas. Concerned professionals often work in multiple centers and need to adapt their practice to an increased flow of patients. This might engage a sense of lack of institutional support and frustration among caregivers. In addition, patients facing shorter consultation lengths might be prone to show aggressive behavior which is known to decrease empathy among healthcare providers. Future research might confirm this result that was also found in one previous study among primary care physicians and nurses [23].

## Conclusion

Empathic mental healthcare providers have lower levels of burnout. Forensic psychiatric staff showed low means of compassionate care and high depersonalization.

This study highlights several perspectives for future research. There is an urgent need for targeted psychosocial interventions to improve the wellbeing and the feeling of achievement among mental healthcare providers. We suggest a potential benefit in the use of psychological interventions, such as meditation and compassion training, to cultivate positive emotions. Integrative models comprising mindfulness, acceptance and value models along with education programs such as Omega should be tailored at an organizational level. To our knowledge, there are no such interventional programs in mental healthcare settings in France.

Further research should be conducted in forensic psychiatric settings. Observational studies are welcomed to assess causal relationships between burnout and empathy and therefore generate the basis for implementing preventive measures.

An essential step in reducing and preventing burnout is to foster attributes and skills such as empathy, resilience, and perception of security. Improving the wellbeing of healthcare providers is the cornerstone of care.

## Limitations

The major limitation of our study is its cross-sectional design which cannot identify causal relationships. Also, the sample was not randomized, and so we cannot rule out the possibility of participation bias. Several potentially significant variables (e.g., personal coping styles, psychotherapy training, supervision) were not included in the survey. Moreover, our results might have been affected by a type II error (failure to detect a real difference) due to the small samples in specific categories (e.g., psychiatric trainees, chief nurses).

Finally, because self-reporting measures are used, desirability can be a factor of bias. However, participation was voluntary, and respondents were informed numerous times of the confidentiality of the study.

## Acknowledgments

We are greatly indebted to the mental health staff involved in this study who filled out the questionnaires. We express our gratitude for their support to Professors Stephane Zuily and Bernard Kabuth of the Lorraine University.

## Conflict of Interest

The authors confirm that there are no conflicts of interest.
